# Non-Coding RNAs as Prognostic Biomarkers: A miRNA Signature Specific for Aggressive Early-Stage Lung Adenocarcinomas

**DOI:** 10.3390/ncrna6040048

**Published:** 2020-12-15

**Authors:** Elisa Dama, Valentina Melocchi, Francesco Mazzarelli, Tommaso Colangelo, Roberto Cuttano, Leonarda Di Candia, Gian Maria Ferretti, Marco Taurchini, Paolo Graziano, Fabrizio Bianchi

**Affiliations:** 1 Cancer Biomarkers Unit, Fondazione IRCCS Casa Sollievo Della Sofferenza, 71013 San Giovanni Rotondo (FG), Italy; e.dama@operapadrepio.it (E.D.); v.melocchi@operapadrepio.it (V.M.); f.mazzarelli@operapadrepio.it (F.M.); t.colangelo@operapadrepio.it (T.C.); r.cuttano@operapadrepio.it (R.C.); 2 Pathology Unit, Fondazione IRCCS Casa Sollievo Della Sofferenza, 71013 San Giovanni Rotondo (FG), Italy; l.dicandia@operapadrepio.it (L.D.C.); p.graziano@operapadrepio.it (P.G.); 3 Thoracic-Surgery Unit, Fondazione IRCCS Casa Sollievo Della Sofferenza, 71013 San Giovanni Rotondo (FG), Italy; gm.ferretti@operapadrepio.it (G.M.F.); m.taurchini@operapadrepio.it (M.T.)

**Keywords:** lung cancer, microRNA, gene expression, biomarkers, prognosis

## Abstract

Lung cancer burden can be reduced by adopting primary and secondary prevention strategies such as anti-smoking campaigns and low-dose CT screening for high risk subjects (aged >50 and smokers >30 packs/year). Recent CT screening trials demonstrated a stage-shift towards earlier stage lung cancer and reduction of mortality (~20%). However, a sizable fraction of patients (30–50%) with early stage disease still experience relapse and an adverse prognosis. Thus, the identification of effective prognostic biomarkers in stage I lung cancer is nowadays paramount. Here, we applied a multi-tiered approach relying on coupled RNA-seq and miRNA-seq data analysis of a large cohort of lung cancer patients (TCGA-LUAD, *n* = 510), which enabled us to identify prognostic miRNA signatures in stage I lung adenocarcinoma. Such signatures showed high accuracy (AUC ranging between 0.79 and 0.85) in scoring aggressive disease. Importantly, using a network-based approach we rewired miRNA-mRNA regulatory networks, identifying a minimal signature of 7 miRNAs, which was validated in a cohort of FFPE lung adenocarcinoma samples (CSS, *n* = 44) and controls a variety of genes overlapping with cancer relevant pathways. Our results further demonstrate the reliability of miRNA-based biomarkers for lung cancer prognostication and make a step forward to the application of miRNA biomarkers in the clinical routine.

## 1. Introduction

The latest global lung cancer data indicate a burden of 2.09 million new cases and 1.76 million deaths in 2018 [1]. The main type of lung cancer is represented by Non-Small-Cell Lung Cancer (NSCLC) (80–85%) including several heterogeneous tumor subtypes: lung adenocarcinoma (ADC, ~40% of lung cancers), squamous cell carcinoma (SqCC, ~25% of lung cancers) and large cell carcinoma (LCC, ~10% of lung cancers) [2]. In the last decades, there have been significant improvements in lung cancer treatment, such as stereotactic ablative radiotherapy (SABR), targeted therapy and immunotherapy [3,4,5]. Nevertheless, despite the successful introduction of these new treatments in clinical practice, global lung cancer mortality rates remained rather unchanged in the last 40 years, with some variability worldwide due to different lifestyle, environmental and occupational exposures [4,6]. However, primary and secondary prevention strategies such as anti-smoking campaigns and the implementation of large CT screening programs resulted in a reduction of lung cancer mortality of ~20% in enrolled patients and progressive lung cancer stage-shift [7,8]. In addition, the high level of molecular heterogeneity of lung cancer enhances the metastatic dissemination of a large fraction of early stage tumors (~30–50%) [9]. In-depth molecular and functional characterization of ADC could help to contextualize tumor heterogeneity in specific molecular subtypes which may suggest alternative therapeutic options. We recently described a 10-gene prognostic signature for stage I ADC which identified a subset of tumors, namely C1-ADC [10,11], with peculiar gene/protein expression and genetic alterations resembling more advanced cancer. This prognostic gene signature can be measured by qRT-PCR, Affymetrix or RNA-seq, in fresh-frozen or in formalin-fixed, paraffin-embedded (FFPE) specimens [11].

To foster clinical translation of this 10-gene signature, here we present a miRNA signature as a surrogate of the 10 genes, for prognostic risk stratification of ADC. A miRNA-based prognostic signature would overcome the problem of using low-quality mRNA when extracted from FFPE samples, which are routinely used for diagnostic purposes. Indeed, shorter non-coding RNA molecules such as miRNA are more resistant to harsh conditions [12,13] and compatible with most of the expression profiling methods including qRT-PCR.

## 2. Results

### 2.1. MiRNA-Signature Identification

**Figure 1:**
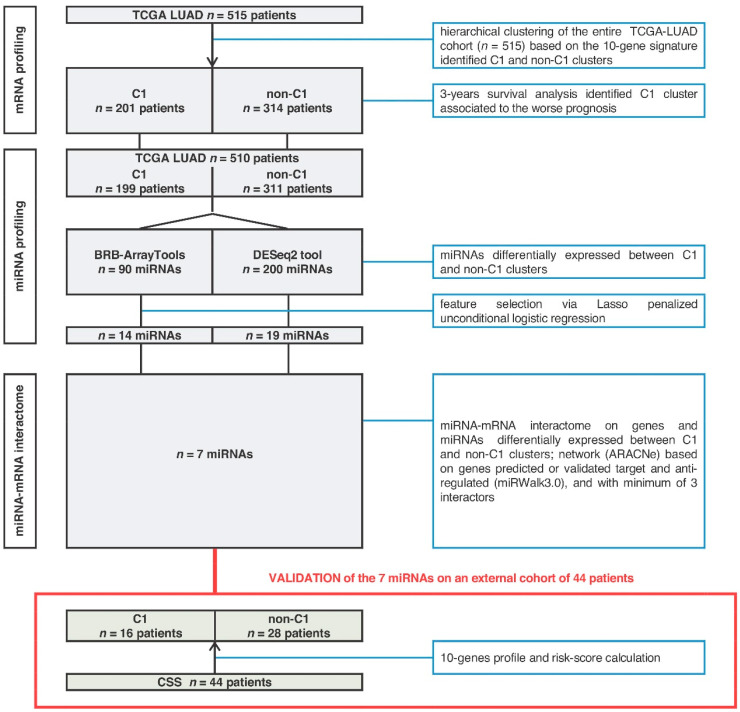
Flow chart of study design with data sets and analysis.

We developed a multi-tiered approach summarized in Figure 1, which allowed us to identify a surrogate miRNA-based signature for prognostication of ADC patients.

Firstly, we performed gene expression profile analysis of a total of 515 ADC patients belonging to the TCGA-LUAD cohort (see Section 4), with available mRNA data. Patients and tumors characteristics are reported in Table 1. Stage I tumors represented 54% of the cohort and smoking habit was present in 71%. Median length of follow-up in survivors was 2.1 years.

**Figure 2:**
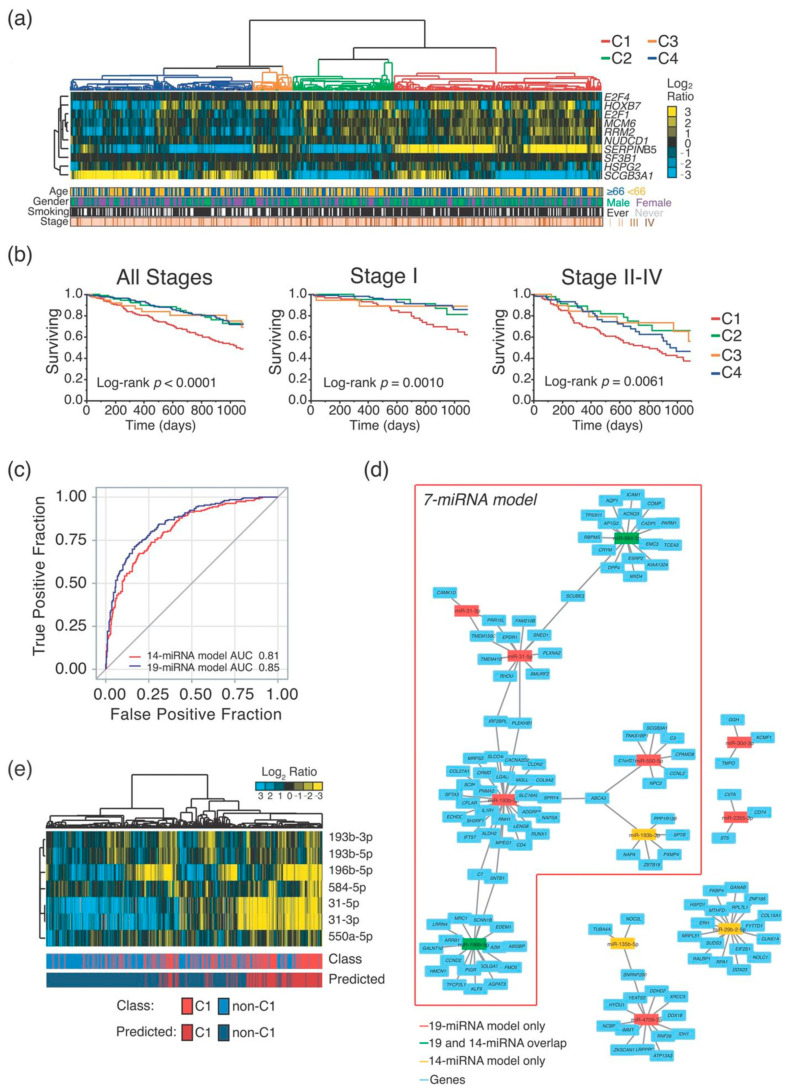
mRNA and miRNA expression profile analysis of the TCGA-LUAD cohort. (**a**) Hierarchical clustering analysis of the 10-gene expression signature. C1-C4 clusters are colored as per the legend. Age, gender, smoking status and stage are colored as per the legend; Unavailable information is colored in white. (**b**) Kaplan–Meier curves for 3-years overall survival stratified by C1–C4 clusters. Log-rank *p*-values are shown for C1 vs. non-C1 clusters (C2-C4) comparison. (**c**) Receiver operating characteristic (ROC) curves showing the False Positive Fraction and True Positive Fraction of the 19- (in blue) and 14-miRNA (in red) models. The areas under curve (AUC) are reported. (**d**) Networks of miRNA derived from 19-, 14- and 7-miRNAs model and corresponding target genes. Light blue rectangles represent genes; red rectangles represent miRNA from 19-miRNA model; yellow rectangles represent miRNA from 14-miRNA model; green rectangles represent miRNA from both 14- and 19-miRNA model. (**e**) Hierarchical clustering of 7-miRNAs in the TCGA-LUAD cohort. C1 and non-C1 tumors (defined according to the 10-gene signature) are colored as per the legend. Predicted C1 and non-C1 tumors (defined according the 7-miRNA logistic model) are colored as per the legend.

Hierarchical clustering analysis using the 10-gene signature of the TCGA-LUAD cohort (*n* = 515) patients revealed 4 main branches, namely C1 (*n* = 201), C2 (*n* = 98), C3 (*n* = 39), and C4 (*n* = 177) clusters (Figure 2a) that are consistent with previous findings [11]. Analysis of the 3-years overall survival showed non-significant differences between C2, C3 and C4 clusters (log-rank test *p*-value = 0.90 and *p*-value = 0.48 in stage I and advanced stages, respectively), that were therefore collapsed into non-C1 clusters. C1 cluster displayed the worse prognosis both in stage I (*p*-value = 0.0010) and in more advanced stages (*p*-value = 0.0061) (Figure 2b). Furthermore, C1 cluster displayed a significant higher fraction of male subjects and patients with more advanced lung cancer, and a nearly significant higher proportion of smokers (Table S1), which is in line with the reported worse prognosis [9].

We then performed miRNA expression profile of 510 out of the 515 ADC of the TCGA-LUAD cohort, with miRNAs expression data available. We used both DESeq2 R package and BRB-ArrayTools (see Section 4) as alternative statistical approaches in order to identify differentially expressed miRNAs in C1 and non-C1 clusters of ADC. We analyzed a total of 382 miRNAs, of which 200 were found differentially expressed by DESeq2 and 90 by BRB-ArrayTools (Table S2A,B, respectively). A total of 87 miRNAs were overlapping in the two sets. Lasso regularization was then applied to identify optimized miRNA-based signatures capable of stratifying C1 from non-C1 tumors. In total, two signatures of 14- (from the 90 miRNA-set) and 19-miRNA (from the 200 miRNA-set) were derived (5 miRNA overlapping; Table 2), which displayed a high accuracy in C1/non-C1 cancer patients stratification (cross-validated AUC = 0.81 and AUC = 0.85, respectively; Figure 2c).

To further reduce complexity of these miRNA-based biomarkers, we looked for a minimal set of miRNAs capable of the same accuracy of the 14- and 19-miRNA signatures to identify C1 aggressive disease. The following assumptions were made: (i) the molecular function of a miRNA is dependent to the network of targeted mRNAs which, in this case, are those differentially expressed in C1/non-C1 tumors; (ii) a prognostic biomarker should be functionally linked to mechanisms involved in tumor progression. Accordingly, we explored the miRNA-mRNA interactome characterizing C1 tumors by performing ARACNe (Algorithm for the Reconstruction of Accurate Cellular Networks) (see Section 4) using the set of 200 miRNA, and a set of 2900 mRNA genes found significantly regulated in C1-ADC (*p*-value < 0.05) by DESeq2 (see Section 4). Our analysis was restricted to genes identified by DESeq2 in order to reduce technical variability. The following rules were applied to rewire C1 miRNA-mRNA interactome: (1) we selected miRNA-mRNA pairs generated in only C1 tumors and specific, but not exclusive, for stage I (*n* = 2858); (2) we selected miRNA predicted to target C1-genes (*n* = 1787, miRWalk3.0, see Section 4), and (3) with an opposite trend of expression than C1-genes (*n* = 598); (4) we selected miRNA interacting with a least three C1-genes (*n* = 528).

**Figure 3:**
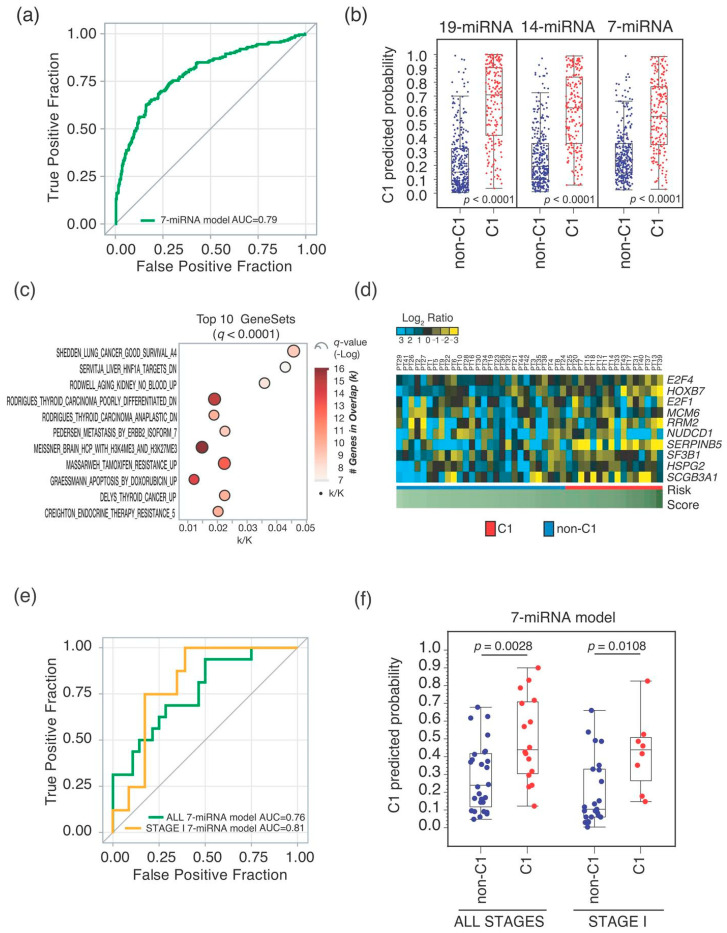
Validation of the 7-miRNA model. (**a**) ROC curve showing the False Positive Fraction and True Positive Fraction of the 7-miRNA model. The AUC is reported. (**b**) Box-plot for C1 predicted probability in C1 and non-C1 patients. Predicted probabilities are calculated through the 19-, 14- and 7-miRNA models. Wilcoxon–Mann–Whitney test *p*-values are reported. (**c**) Bubble plot of top 10 GeneSets found significantly overlapping with gene networks targeted by the 7-miRNA signature. Bubbles size is proportional to statistical significance (-Log of *q*-value) and color codes refer to number of genes found in the overlap. In *X*-axis, ratios (k/K) of overlap of the query set of genes (k) with overlapping GeneSet size (K). (**d**) Heatmap of the 10-gene expression of CSS cohort. C1 and non-C1 tumors are colored as per the legend. Risk scores are calculated based on the 10-gene risk model. (**e**) ROC curves showing the False Positive Fraction and True Positive Fraction of the 7-miRNA model in the CSS cohort, for all stages (in green) or only stage I tumors (in orange). The AUC are reported. (**f**) Box-plot for C1 predicted probability in C1 and non-C1 tumors in CSS cohort, for all-stages tumors and stage I tumors. Predicted probabilities are calculated through the 7-miRNA model. Wilcoxon–Mann–Whitney test *p*-values are reported.

Among the miRNA-mRNA networks identified, we found a set of interacting networks with 7 miRNA as “HUBs” which derived from both the 19-miRNA and 14-miRNA signatures (Table 2 and Figure 2d). Hierarchical clustering analysis of this 7-miRNA signature (Table S3) showed an overall increased expression in the more aggressive C1 tumors (Figure 2e). Importantly, the 7-miRNA signature had a cross-validated AUC of 0.79 in C1/non-C1 patients’ stratification, which is comparable to the other two signatures (Figure 3a), as well as when we considered differences in C1 predicted probability (Figure 3b). The predicted C1 class from all the three signatures (7-, 14- and 19-miRNA) presented significantly increased hazard of death at 3 years in patients of all stages, with an increased risk comparable to C1 patients identified by using the 10-gene signature (Table 3). However, when we focused the analysis to stage I ADC patients, we scored that the best risk-stratification was held by the 7-miRNA signature with approximately two-fold increased risk of death for C1 patients (HR = 2.11; 95% Confidence Interval: 1.11–4.00; *p*-value = 0.0223) (Table 3). Interestingly enough, the networks of genes targeted by these 7 miRNAs were found significantly (*q*-value < 0.0001) enriched in gene sets representing molecular mechanisms related to cancer progression, which fulfilled our initial hypotheses (Figure 3c).

Despite most of 90 miRNAs identified by BRB-ArrayTools (87/90, 97%) were comprised in the 200-miRNA set found by DESeq2, including 12 out of 14 miRNAs of the BRB-derived model, we performed ARACNe as well by using this 90-miRNAs set. Among the three not overlapping miRNAs, only hsa-miR-210-3p passed all the selection filters we described previously. However, when we added this additional miRNA to the 7-miRNA signature and performed cross-validation in C1/non-C1 patients’ stratification, the prediction performance remained the same (AUC = 0.79).

### 2.2. Seven-miRNA-Signature Validation

Finally, we validated the 7 miRNA-signature in an external cohort of 44 lung adenocarcinoma patients, which was collected at the IRCCS Casa Sollievo della Sofferenza Hospital (CSS). Clinical pathological characteristics of the CSS cohort are reported in Table 1, with an overrepresentation of stage I tumors in CSS (70%) with respect to the TCGA-LUAD cohort (54%). We performed qRT-PCR analysis of FFPE samples using the 10-gene signature and calculated relative risk-score to stratify the cohort into C1 (*n* = 16) and non-C1 (*n* = 28) groups (Figure 3d) (see Section 4). Next, we performed Low-Density Taqman miRNA Arrays to profile the 7-miRNA signature in the same cohort of 44 ADC and, using logistic regression, we rederived a model based on the expression profile of the 7-miRNA signature (Table S4). The 7-miRNA model stratified C1 from non-C1 tumors with an AUC of 0.76 (Figure 3e) and with significant difference (*p*-value = 0.0028) in C1 predicted probability (Figure 3f). Remarkably, when we limited the analysis to stage I tumors, we scored an AUC of 0.81 (Figure 3e) and a significant difference (*p*-value = 0.0108) in C1 predicted probability (Figure 3f).

## 3. Discussion

Improvements in lung cancer early diagnosis by large scale low-dose CT screening trials is resulting in a stage-shift towards earlier stage lung cancer, with subsequent reduction in mortality as observed in NELSON and NLST trials [7,8]. For this reason, there is an urgent need of prognostic biomarkers for patients with stage I lung cancer who could eventually benefit from systemic adjuvant chemotherapy (platinum-based) rather than molecular targeted/immuno therapeutics in case of aggressive disease. Nowadays, the advent of more sophisticated and precise bioinformatic tools and computational approaches has sped up the identification of novel diagnostic and prognostic cancer biomarkers, either focused on proteins, coding transcripts, epigenome modifications or non-coding transcripts [14,15]. In lung cancer, in particular, the differential expression of miRNAs allowed the development of innovative and promising cancer biomarkers [16].

Here we present surrogate miRNA signatures which recapitulate a previously described 10-gene prognostic signature in stage I ADC [11]. The 7-, 14- and 19-miRNA signatures were all effective in identifying aggressive C1-ADC disease (AUC = 0.79–0.85). Notably, 6 out of 7 miRNAs of the 7-miRNA signature were well-detected in FFPE samples (median Ct < 30; Table S3) which confirmed the proven higher stability of miRNAs in low-quality RNA [17]. Importantly, in our approach, we adopted a network-rewiring strategy by specifically selecting miRNA-mRNA pairs which characterize aggressive stage I tumors (C1). Such an approach allowed us to select a core of 7 miRNAs capable to stratify C1 from non-C1 samples with an accuracy comparable to the 14- and 19-miRNA models (Figure 2c and Figure 3a), and, importantly, interacting with C1 tumor transcriptome. This is relevant for capturing molecular mechanisms controlled by miRNAs which are associated to tumor progression. As a matter of fact, we observed a large overlap between the ‘7-miRNA network’ with several gene sets representing cancer relevant pathways (Figure 3c). Further experiments are therefore warranted in order to investigate whether this 7-miRNA network is functionally linked to lung cancer progression.

The 7-miRNA signature is composed by hsa-miR-31-5p, hsa-miR-31-3p, hsa-miR-193b-3p, hsa-miR-193b-5p, hsa-miR-196b-5p, hsa-miR-550a-5p and hsa-miR-584-5p. Some of these miRNAs were described to be altered in cancer and also with a functional role. Alterations in the expression of miR-31-5p and miR-31-3p were associated with a variety of cancers including lung cancer and have both oncogenic and onco-suppressor behavior [18,19,20,21,22,23,24]. Furthermore, hsa-miR-193b-3p was found associated to a tumor-suppressor phenotype (by targeting *STMN1*) in hepatocellular carcinoma [25] and colorectal cancer [26]. Remarkably, hsa-miR-193 in prostate cancer was shown to target *FOXM1* and *RRM2* [27], the last being one of the genes composing the prognostic 10-gene signature. The hsa-miR-196b-5p was found to promote tumor progression in non-small cell lung cancer when up-regulated [28]. Interestingly, hsa-miR-196b-5p was also found to molecularly interact with *HOXB7* and *GALNT5* and inhibit their expression in colorectal cancer [29]. Again, *HOXB7* is one of the genes composing the 10-gene signature, which we also recently showed to promote a stem cell-like phenotype in lung adenocarcinoma [30]. On the other hand, very little is known about hsa-miR-550a-5p and hsa-miR-584-5p biological functions. However, hsa-miR-550a-5p was found to be a prognostic factor in ADC in association with other 3 miRNAs indicating its possible oncogenic role [31]. Lastly, in gastric cancer, hsa-miR-584-5p was found to induce apoptosis and inhibits proliferation [32], while in hepatocellular carcinoma hsa-miR-584-5p was shown to have an oncogenic role [33].

In conclusion, we developed a 7-miRNA signature which is capable in identifying aggressive early stage lung adenocarcinoma. The expression profile of such miRNAs can be measured by standard qRT-PCR and using FFPE samples. A limit of the present study is the relatively small size of the external validation cohort (CSS) and the short follow-up (1.6 median years in survivors, and three deaths recorded within 3 years), which did not allow us to quantify the excess of mortality risk for patients with predicted aggressive tumors.

## 4. Materials and Methods

### 4.1. TCGA-LUAD Cohort

We selected the cohort of 515 patients with lung adenocarcinomas from the TCGA data portal (https://portal.gdc.cancer.gov/) at 2018. A total of 510 tumors were profiled for both gene and miRNA expression. Log2 read counts were used for expression analysis. Patients follow-up information was used for survival analysis: overall survival was defined as the time from the date of tumor resection until death from any cause. Follow-up was truncated at 3 years to reduce the potential overestimation of overall mortality with respect to lung cancer–specific mortality.

### 4.2. The CSS Cohort

We selected a cohort of 44 patients with lung adenocarcinoma underwent surgery between February 2017 and February 2020 at the CSS. Written informed consent was obtained from all study patients. None of these patients received preoperative chemotherapy. Clinical information was obtained through review of medical records. Vital status was assessed through the Vital Records Offices of the patients’ towns of residence or by contacting directly the patients or their families. 

### 4.3. Gene Expression Analysis of the TCGA-LUAD Cohort

Hierarchical clustering analysis was performed on the 10-gene signature for the entire cohort of 510 patients. Clustering was done by using Cluster 3.0 for Mac OS X (C Clustering Library 1.56, Tokyo, Japan) with uncentered correlation and centroid linkage, and Java TreeView software environment (version 1.1.6r4; http://jtreeview.sourceforge.net). A total of four main branches were selected to build clusters. Kaplan–Meier survival curves were stratified by clusters and log-rank test *p*-values were calculated. C1 cluster was associated to the worse prognosis, and all other clusters were pooled together (non-C1 clusters).

To retain most informative expression data (i.e., transcripts detected in most of tumor samples), we considered miRNAs with raw counts >0 in at least the 50% of patients either in C1 or non-C1, identifying a total of 382 miRNAs. This allowed us to reduce also the complexity of the TCGA-LUAD dataset (2237 miRNAs). We applied the complexity reduction also to genes and we selected the most varying across all samples (standard deviation in the top 25%), identifying a total of 4899 genes. Using DESeq2 R package (R Core Team, R Foundation for Statistical Computing, Vienna, Austria) [34], we identified a total of 2900 differentially expressed genes between C1 and non-C1 tumors.

BRB-ArrayTools [35] and DESeq2 (R package) [34] tools were used for class prediction (C1 cluster vs. non-C1 clusters) according to miRNA expression. BRB-ArrayTools uses statistics based on two-sample T-test with multivariate permutations test (1000 random permutations); confidence level of false discovery rate assessment, 80%; maximum allowed proportion of false-positive genes, 0.05. DESeq2 is based on Wald test statistics to identify differentially expressed transcripts. Lists of miRNAs differentially expressed were obtained from BRB-ArrayTools and DESeq2 tools were subsequently reduced via Lasso regularization. In details, a penalized unconditional logistic regression was applied considering cluster as discrete outcome (C1 cluster vs. non-C1 clusters) and miRNA expressions as explanatory variables. Cross-validated (10-fold) log-likelihood with optimization (50 simulations) of the tuning penalty parameter was used to control for potential overfitting.

Starting from differentially expressed genes (identified with DESeq2) and miRNAs (identified with both DESeq2 and BRB-ArrayTools), we used ARACNe [36] with 1000 bootstraps to infer direct regulatory relationships between transcriptional regulators (i.e., miRNAs) and target genes. ARACNe was performed using all patients, stage I patients and stage II-IV patients. miRNA target genes were retrieved using miRWalk 3.0 [37].

Probability of being in the C1 cluster was estimated using the unconditional logistic regression for the three signatures of 19, 14 and 7 miRNAs. Model performance was assessed using the cross-validated area under the receiver operating curve, and assessing the difference in C1 predicted probability between C1 and non-C1 patients (Wilcoxon–Mann–Whitney test). Cox regression model was used to evaluate the prognostic role of these miRNA signatures and their ability to recapitulate the risk-stratification of the original 10-genes signature.

To receive insights into the biology of the 7-miRNA model, we verified the enrichment of cancer-relevant pathways associated to their target genes. We investigated the Molecular Signature Database (MSigDB; v7.2) (https://www.gsea-msigdb.org/gsea/msigdb/annotate.jsp) using the list of 87 targeted genes by interrogating the CGP (chemical and genetic perturbations, 3358 gene sets). Bubble plot analysis was performed using JMP 15.2.1 (SAS Institute, Inc., Cary, NC, USA) software.

Hierarchical clustering analysis was performed on the 7-miRNA signature for 510 patients, those with available miRNA expression data. Clustering was completed by using Cluster 3.0 for Mac OS X (C Clustering Library 1.56) with uncentered correlation and centroid linkage, and Java TreeView software environment (version 1.1.6r4; http://jtreeview.sourceforge.net).

### 4.4. RNA Extraction and qRT-PCR Analysis and Data Interpretation

One tissue core (1.5 mm in diameter) from FFPE blocks, in representative tumor areas with adequate tumor cellularity (>60%) selected by a pathologist, was processed for total RNA extraction. The AllPrep DNA/RNA FFPE kit (QIAGEN, Inc., Hilden, Germany) was used for total RNA extraction. Quantitative real-time PCR (qRT-PCR) was performed to analyze the 10-genes signature as described in Dama et al. [11]. Briefly, RNA was quantified using Nanodrop ND-10000 Spectrophotometer and a total of 200 ng was retro-transcribed using SuperScript VILO cDNA Synthesis Kit (Thermo Fisher Scientific, Inc., Waltham, MA, USA) (Thermo Fisher Scientific) and pre-amplified for 10 cycles with PreAmp Master Mix Kit (Thermo Fisher Scientific), following manufacturer’s instructions. qRT-PCR analysis was performed starting from 1:10 diluted pre-amplified cDNA, using the TaqMan Fast Advance Master Mix and hydrolysis probes (Thermo Fisher Scientific; for primers see Dama et al. [11]), in a QuantStudio 12k Flex (Thermo Fisher Scientific). Thermal cycling amplification was performed with an initial incubation at 95 °C for 30 s, followed by 45 cycles of 95 °C for 5 s and 60 °C for 30 s. For miRNA expression analysis, a total of 10 ng RNA was reverse-transcribed using the TaqMan Advanced miRNA cDNA Synthesis Kit (Thermo Fisher Scientific). Poly(A) tailing, adapter ligation, RT reaction and miR-Amp were performed following manufacturer’s instructions. qRT-PCR was performed following manufacturer’s instructions (i.e., 95 °C for 30 s, 45 cycles of 95 °C for 5 s, and 60 °C for 30 s) using a Card Custom Advance (Thermo Fisher Scientific; Table S5) in a QuantStudio 12k Flex (Thermo Fisher Scientific). The hsa-miR-16-5p was used as standard reference for CT normalization using a previously described methodology [38]. Briefly, the normalized CT of each miRNA (i) of each sample (j) was calculated as the difference between the raw CT_ij_ and a scaling factor (SF) specific for each sample (j); the SF_j_ represented the difference between the raw CT of the miRNA “hsa-miR-16-5p” used as a reference in the sample (j) and a constant equal to 21.87. Notably, hsa-miR-16-5p expression profile analysis in both TCGA-LUAD and CSS cohorts revealed a comparable expression in C1 and non-C1 tumors subsets (Table S6).

Risk-scores were assigned to each patient based to the 10-gene risk model described in Dama et al. [11]. Before applying the risk-model, data were rescaled (q1-q3 normalization). Patients with risk-scores higher than the 66th percentiles [11] were classified as C1 tumors. Next, unconditional logistic regression (C1 vs. non-C1 tumors) with 7 miRNAs as explanatory variables was applied, and the area under the receiver operating curve was calculated. Difference in C1 predicted probability between C1 and non-C1 patients was evaluated through Wilcoxon–Mann–Whitney test.

All statistical analyses were performed using SAS software, version 9.4 (SAS Institute, Inc., Cary, NC, USA) and R 3.3.1 (R Core Team, 2016) and JMP 15 (SAS). *p*-values less than 0.05 were considered statistically significant.

## Figures and Tables

**Table 1:** Patients and tumors characteristics.

	TCGA-LUAD Cohort *n* = 515	CSS Cohort *n* = 44
**Age [years]**		
Median (Q1; Q3)	66 (59;73) ^1^	73 (67;77)
**Gender**		
Male	238 (46.2%)	27 (61.4%)
Female	277 (53.8%)	17 (38.6%)
**Smoking status**		
Current/former smoker	367 (71.3%)	20 (45.5%)
Never smoker	63 (12.2%)	11 (25.0%)
Missing smoking status	85 (16.5%)	13 (29.5%)
**Stage**		
Stage I	279 (54.2%)	31 (70.5%) ^2^
Stage II	124 (24.1%)	6 (13.6%)
Stage III	84 (16.3%)	6 (13.6%)
Stage IV	27 (5.2%)	1 (2.3%)
Missing stage	1 (0.2)	-
**Follow-up ^3^**		
Survivors length of follow-up		
<1 year	52 (10.3%)	13 (31.7%)
1–2 years	128 (25.3%)	11 (26.8%)
2–3 years	56 (11.1%)	10 (24.4%)
>3 years	133 (26.3%)	5 (12.2%)
Deaths within 3 years	137 (27.1%)	2 (4.9%) ^4^

Percentages could not add up to 100 due to rounding; ^1^ 19 patients with missing information on age; ^2^ 1 patient with adenocarcinoma in situ; ^3^ 9 patients with missing follow-up in the TCGA-LUAD cohort; ^4^ 3 deaths were excluded: 1 without date of death, and 2 within 30 days from surgery.

**Table 2:** TCGA-LUAD cohort. Differentially expressed miRNAs composing the three signatures with 19, 14 and 7 miRNAs.

miRNA	Accession	Signature	TCGA-LUAD Cohort— C1 vs. non-C1 Cluster		
			FC	*p*-value ^1^	C1 Trend
hsa-miR-193b-5p	MIMAT0004767	19- and 7-miRNA	1.5	3.3 × 10^−7^	↑
hsa-miR-31-3p	MIMAT0004504	19- and 7-miRNA	3.2	1.9 × 10^−20^	↑
hsa-miR-31-5p	MIMAT0000089	19- and 7-miRNA	3.1	1.7 × 10^−18^	↑
hsa-miR-550a-5p	MIMAT0004800	19- and 7-miRNA	1.5	6.0 × 10^−9^	↑
hsa-miR-196b-5p	MIMAT0001080	19-, 14-miRNA and 7-miRNA	3.2	9.8 × 10^−21^	↑
hsa-miR-584-5p	MIMAT0003249	19-, 14-miRNA and 7-miRNA	2.8	1.2 × 10^−40^	↑
hsa-miR-30d-5p	MIMAT0000245	19- and 14-miRNA	0.6	4.8 × 10^−16^	↓
hsa-miR-582-3p	MIMAT0004797	19- and 14-miRNA	2.2	2.5 × 10^−18^	↑
hsa-miR-9-5p	MIMAT0000441	19 and 14-miRNA	1.8	1.7 × 10^−6^	↑
hsa-let-7c-3p	MIMAT0026472	19-miRNA	0.8	1.9 × 10^−2^	↓
hsa-miR-138-5p	MIMAT0000430	19-miRNA	1.9	1.2 × 10^−10^	↑
hsa-miR-196a-5p	MIMAT0000226	19-miRNA	1.4	2.7 × 10^−2^	↑
hsa-miR-203a-3p	MIMAT0000264	19-miRNA	1.4	3.1 × 10^−4^	↑
hsa-miR-215-5p	MIMAT0000272	19-miRNA	5.0	1.2 × 10^−37^	↑
hsa-miR-2355-3p	MIMAT0017950	19-miRNA	1.3	5.4 × 10^−5^	↑
hsa-miR-30d-3p	MIMAT0004551	19-miRNA	0.6	2.5 × 10^−15^	↓
hsa-miR-4709-3p	MIMAT0019812	19-miRNA	0.5	1.3 × 10^−19^	↓
hsa-miR-548b-3p	MIMAT0003254	19-miRNA	0.6	7.2 × 10^−10^	↓
hsa-miR-675-3p	MIMAT0006790	19-miRNA	2.1	1.5 × 10^−8^	↑
hsa-miR-193b-3p	MIMAT0002819	14- and 7-miRNA	1.4	8.6 × 10^−6^	↑
hsa-miR-135b-5p	MIMAT0000758	14-miRNA	0.7	3.7 × 10^−6^	↓
hsa-miR-187-3p	MIMAT0000262	14-miRNA	0.6	2.3 × 10^−4^	↓
hsa-miR-192-5p	MIMAT0000222	14-miRNA	3.1	9.8 × 10^−21^	↑
hsa-miR-210-3p	MIMAT0000267	14-miRNA	1.2	6.4 × 10^−2^	↑
hsa-miR-29b-2-5p	MIMAT0004515	14-miRNA	0.7	1.2 × 10^−7^	↓
hsa-miR-3065-3p	MIMAT0015378	14-miRNA	0.7	4.2 × 10^−5^	↓
hsa-miR-375-3p	MIMAT0000728	14-miRNA	1.2	1.7 × 10^−1^	↑
hsa-miR-708-5p	MIMAT0004926	14-miRNA	1.3	2.7 × 10^−3^	↑

^1^ Wald test adjusted (Benjamini–Hochberg method) from DESeq2 tool. Accession, miRbase mature miRNA accession number. FC, fold change. C1 trend, expression trend in C1 samples versus non-C1 samples.

**Table 3:** TCGA-LUAD cohort. Univariate and multivariable Cox regression analyses for 3-years overall survival in patients of all stages and stratified by stage.

		Univariate Analysis		Multivariable Analysis ^1^	
	*n* (*n* Deaths)	HR (95% CI)	Wald Test *p*-value	HR (95% CI)	Wald Test *p*-value
**ALL STAGES**	**501 (135) ^2^**				
10-gene	194 (75)	2.21 (1.57–3.10)	<0.0001	2.03 (1.43–2.87)	<0.0001
19-miRNA	169 (66)	2.13 (1.52–2.99)	<0.0001	1.85 (1.31–2.61)	0.0005
14-miRNA	165 (67)	2.17 (1.55–3.04)	<0.0001	2.06 (1.46–2.91)	<0.0001
7-miRNA	146 (67)	2.90 (2.07–4.06)	<0.0001	2.69 (1.91–3.78)	<0.0001
**STAGE I**	**274 (40)**				
10-gene	92 (23)	2.86 (1.53–5.36)	0.0010	2.96 (1.55–5.65)	0.0010
19-miRNA	73 (11)	1.07 (0.54–2.15)	0.8462	1.12 (0.55–2.26)	0.7529
14-miRNA	79 (17)	1.90 (1.01–3.56)	0.0451	1.99 (1.05–3.79)	0.0359
7-miRNA	65 (15)	2.11 (1.11–4.00)	0.0223	2.14 (1.11–4.12)	0.0235
**STAGE II-IV**	**226 (95)**				
10-gene	101 (52)	1.69 (1.13–2.54)	0.0108	1.64 (1.08–2.49)	0.0207
19-miRNA	95 (55)	2.27 (1.51–3.41)	<0.0001	2.18 (1.43–3.31)	0.0003
14-miRNA	86 (50)	2.00 (1.34–3.00)	0.0007	2.04 (1.35–3.08)	0.0007
7-miRNA	80 (52)	2.89 (1.93–4.33)	<0.0001	2.91 (1.93–4.39)	<0.0001

^1^ all stages analyses were adjusted for age, sex, smoking status and stage; analyses stratified by stage were adjusted for age, sex and smoking status; ^2^ 1 patient with missing stage and 9 patients with missing follow-up.

## Supplementary Materials

The following are available online at https://www.mdpi.com/2311-553X/6/4/48/s1. Table S1: Patients and tumors characteristics according to C1/non-C1 stratification; Table S2A: TCGA-LUAD cohort. *n* = 200 miRNAs significantly regulated by DESeq2 in C1 vs. non-C1 clusters comparison; Table S2B. TCGA-LUAD cohort. *n* = 90 miRNAs significantly regulated by BRB-ArrayTools in C1 vs. non-C1 clusters comparison; Table S3: distributions of expression for the 7 miRNAs (median, first-Q1 and third-Q3 quartiles are reported) in TCGA-LUAD and CSS cohorts; Table S4: Logistic model regression coefficients for the 7 miRNAs, for all stages and stage I tumors of CSS cohort; Table S5: TaqMan assays used to amplify miRNA of the 7-miRNA signature, the “standard reference” miR-16-5p is also reported; Table S6: Expression profile of miR-16-5p in the CSS and TCGA-LUAD cohorts (median, first-Q1 and third-Q3 quartiles are reported).
